# Serum human epididymis protein 4 and long-term mortality in U.S. women: a nationally representative cohort study

**DOI:** 10.1016/j.clinsp.2026.101133

**Published:** 2026-09-11

**Authors:** Yu-Wei Fang, Hsuan-Cheng Lin, Chikang Wang, Chien-Yu Lin

**Affiliations:** a Division of Nephrology, Department of Internal Medicine, Shin-Kong Wu Ho-Su Memorial Hospital, Taipei, Taiwan; b School of Medicine, College of Medicine, Fu Jen Catholic University, New Taipei, Taiwan; c School of Medicine, College of Medicine, Taipei Medical University, Taipei City, Taiwan; d Department of Environmental Engineering and Health, Yuanpei University of Medical Technology, Hsinchu, Taiwan; e Department of Internal Medicine, En Chu Kong Hospital, New Taipei City, Taiwan

**Keywords:** Human epididymal secretory protein E4 (HE4), National health and nutrition examination survey (NHANES), Cancer mortality, Chronic lower respiratory disease mortality, Women

## Abstract

**Purpose:**

Serum Human Epididymal secretory protein E4 (HE4) is a biomarker in gynecologic oncology and is increasingly linked to fibrosis and adverse outcomes across organ systems; however, its population-level prognostic value beyond cancer and advanced organ failure remains unclear.

**Patients and methods:**

We analyzed women aged ≥ 20-years in the National Health and Nutrition Examination Survey (NHANES) 2001–2002 cycle with serum HE4 (unweighted n = 2200; weighted population ≈46.9 million) linked to mortality outcomes through 2019.

**Results:**

Over a median 199.3-months of follow-up, 466 deaths occurred. Higher ln-HE4 levels were strongly associated with Chronic Kidney Disease (CKD) (Odds Ratio [OR = 2.07], 95% CI: 1.38–3.12) and history of lung disease (OR = 1.34, 95% CI: 1.06–1.71). In fully adjusted models, higher ln-HE4 was associated with all-cause mortality (Hazard Ratio [HR = 1.29], 95% CI: 1.03–1.63) and cancer mortality (HR = 1.60, 95% CI: 1.02–2.49). Chronic lower respiratory disease mortality showed a positive but imprecise association because of limited events (n = 25; HR = 3.16, 95% CI: 1.26–7.91). For diabetes mortality, the estimate was also imprecise and not statistically significant (n = 18; HR = 3.84, 95% CI: 0.92–16.00). Trends across quartiles were significant for all-cause (p for trend = 0.001) and cancer mortality (p for trend = 0.006). Sensitivity analyses were broadly consistent, although estimates for outcomes with few events should be interpreted cautiously.

**Conclusions:**

In a nationally representative cohort of U.S. women, higher serum HE4 was associated with increased risks of all-cause and cancer mortality. Associations with chronic lower respiratory disease and diabetes mortality were based on few events and should be interpreted cautiously. These results support HE4 as a potential population-level prognostic biomarker beyond oncology, warranting validation in other cohorts and mechanistic studies.

## Highlights

• Serum HE4 was evaluated as a mortality biomarker in U.S. women using NHANES data.
• Higher HE4 was linked to chronic kidney disease and history of lung disease.
• Elevated HE4 predicted higher all-cause, cancer, and respiratory mortality risks.
• Dose-response trends confirmed rising mortality with increasing HE4 quartiles.
• HE4 may serve as a population-level prognostic marker beyond oncology settings.

## Graphical abstract

**Figure:**
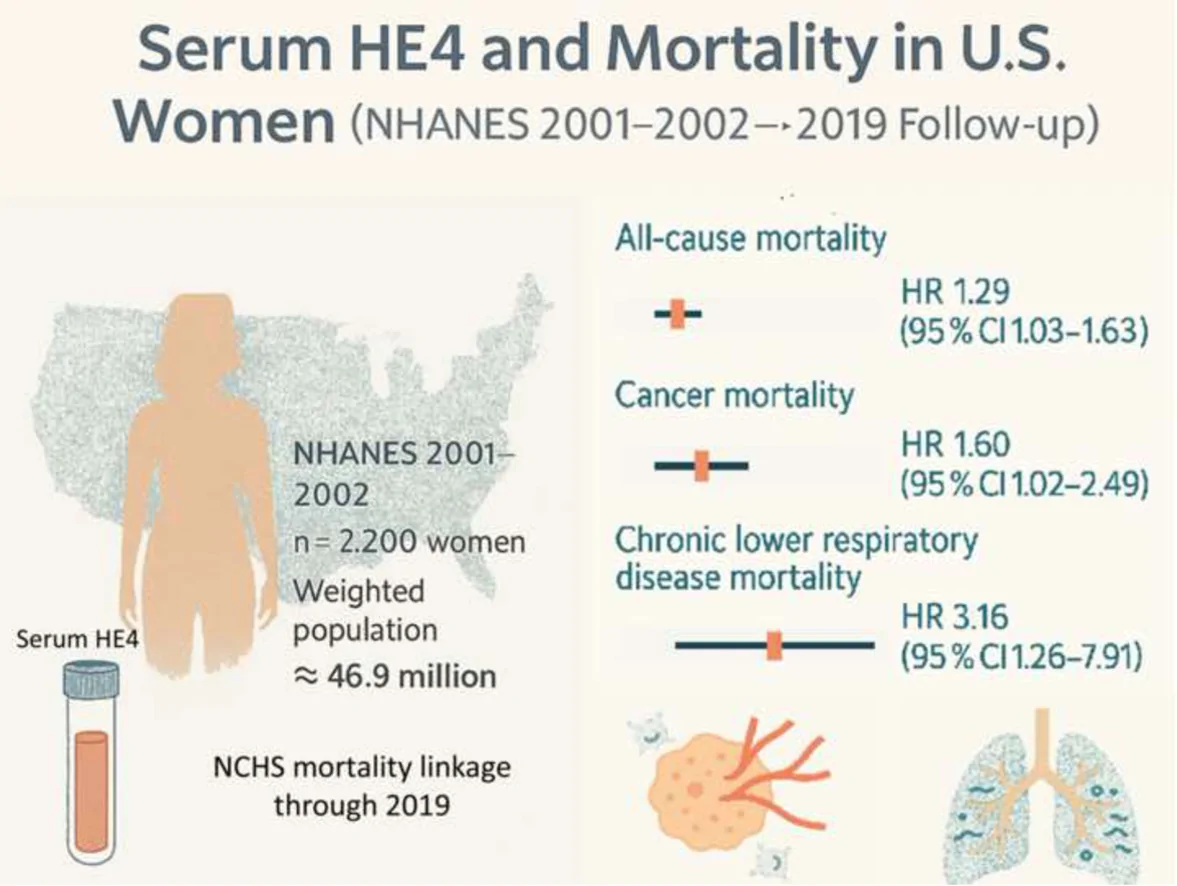

## Introduction

Human Epididymis protein-4 (HE4) is a glycoprotein secreted extracellularly and structurally defined by a four-disulfide core motif typical of the whey acidic protein family.^1^ Through the activation of STAT3 signaling, HE4 enhances the expression of angiogenesis-related mediators and diminishes anti-tumor immune infiltration, thereby generating a tumor milieu that favors both new vessel formation and immune tolerance.^2^ HE4 was originally discovered as an epididymis-specific transcript but later recognized for its overexpression in epithelial ovarian cancer. In oncology, HE4 has been widely investigated for both early diagnosis and prognosis of ovarian and endometrial cancers. Compared with Cancer Antigen 125 (CA125), HE4 offers higher specificity in distinguishing malignant from benign gynecological conditions.^3^ Moreover, combining HE4 with CA125 further improves diagnostic accuracy^4^ and HE4 provides additional prognostic information regarding tumor aggressiveness and survival outcomes.^5^ HE4 has also been explored in various biological fluids (e.g., urine, bronchoalveolar lavage fluid, ascites), further broadening its diagnostic and prognostic potential. For example, urinary HE4 has shown promise in early detection of ovarian cancer and recurrence monitoring, sometimes preceding serum biomarker changes in resistant cases and its application.^6^

Over time, HE4 has expanded from oncology to systemic diseases. HE4 also functions as a serine protease inhibitor, preventing extracellular matrix degradation and inhibiting matrix metalloproteinase activity, thereby promoting fibrotic remodeling.^7^ Elevated HE4 concentrations have been linked to renal fibrosis,^8^ hepatic fibrosis in autoimmune liver disease,^9^ and airway remodeling in chronic obstructive pulmonary disease.^10^ Beyond fibrosis, HE4 has emerged as a prognostic biomarker in cardiovascular and pulmonary disorders. In patients with acute heart failure and chronic kidney disease, serum HE4 independently predicted rehospitalization and cardiovascular mortality.^11^ Similarly, in chronic heart failure, HE4 levels increased with disease severity and independently predicted long-term mortality and rehospitalization.^12^ Elevated HE4 has also been observed in idiopathic pulmonary arterial hypertension, where it predicted clinical worsening and poorer survival.^13^ Furthermore, in critically ill patients with coronavirus disease 2019, higher admission HE4 levels were strongly associated with in-hospital mortality.^14^ Collectively, these findings suggest that HE4 extends beyond oncology, serving as a biomarker of fibrosis, disease progression, and adverse outcomes in diverse clinical contexts.

Although HE4 has been widely studied as a biomarker in oncology and in patients with cardiovascular, pulmonary, and renal diseases, its role outside cancer and advanced organ failure remains poorly characterized. Moreover, evidence is limited on whether HE4 is independently associated with long-term mortality in population-based cohorts, beyond established clinical and biochemical risk factors. To address these knowledge gaps, we investigated the associations between serum HE4 and both all-cause and cause-specific mortality in a nationally representative sample of U.S. adults. Leveraging data from the National Health and Nutrition Examination Survey (NHANES) with extended follow-up, our study aimed to examine whether serum HE4 is independently associated with all-cause and cause-specific mortality in U.S. women, using nationally representative NHANES data with long-term follow-up.

## Materials and methods

### Study population

**Fig. 1:**
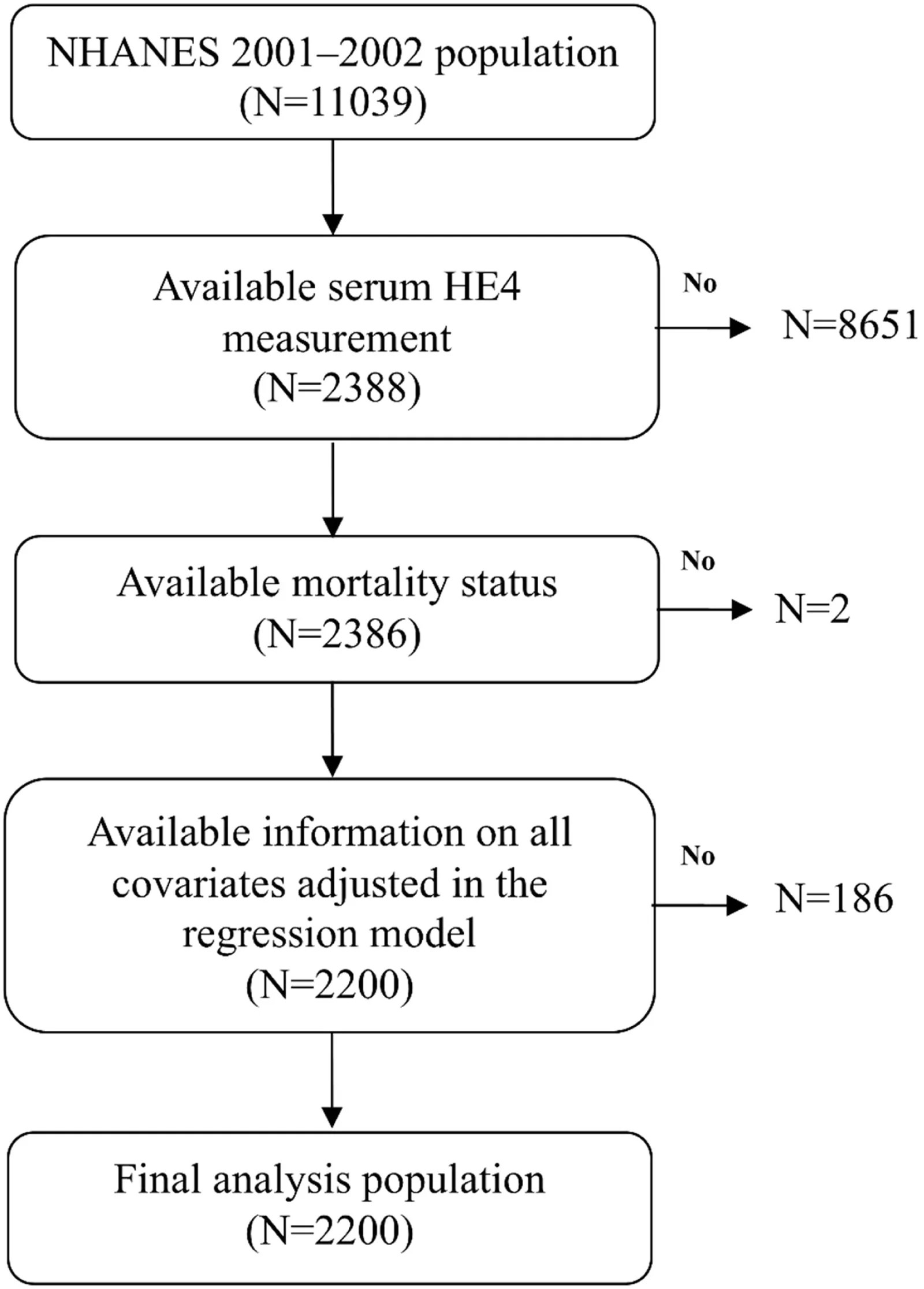
Flow chart algorithm.

This nationally representative cohort study was reported in accordance with the Strengthening the Reporting of Observational Studies in Epidemiology (STROBE) guidelines for observational studies. The NHANES is a continuous program conducted biennially to capture a nationally representative picture of non-institutionalized U.S. residents. Using a complex, multistage probability sampling framework, the survey provides reliable estimates that can be generalized to the broader U.S. population. The NHANES protocols for 2001‒2002 were approved by the NCHS Research Ethics Review Board (Protocol #98–12), and all participants provided written informed consent. This secondary analysis used publicly available, de-identified data and was granted exemption from further institutional review by the NCHS Research Ethics Review Board. Comprehensive details regarding data collection procedures and informed consent are publicly available on the NHANES website.^15^ For the present analysis, data from the 2001–2002 cycle were examined, beginning with 11,039 participants. Among them, 2388 women had valid serum HE4 measurements. After further excluding participants without mortality information (n = 2) and those lacking complete covariate data required for regression modeling (n = 186), the final analytic cohort comprised 2200 individuals (unweighted n = 2200; weighted population 46,904,119). The overall sampling and exclusion process is summarized in Fig. 1.

### Measurement of serum HE4

Serum HE4 data were obtained from surplus specimens collected in the NHANES 2001–2002 cycle, restricted to women aged ≥ 20-years who had provided consent for future research use. All assays were performed following standardized operating procedures. HE4 concentrations were measured using an electrochemiluminescence immunoassay (Meso Scale Discovery, Rockville, MD, USA). Quality assurance included replicate quality-control pools analyzed on each assay plate, with plates repeated if the coefficient of variation exceeded 25%. The analytic range of the assay was 0.55–3600 picomolar, with a lower limit of detection of 0.006 picomolar. In this study, all observed values were above the detection limit, and thus no substitution was required. All samples were assayed without dilution; specimens below the 1st percentile or above the 99th percentile were reanalyzed for confirmation, and entire plates were repeated if systematic bias was detected. Only results passing quality-control criteria were released. Detailed analytic methods are available on the NHANES website.^16^

### Covariates

Data on demographic and lifestyle characteristics were derived from NHANES questionnaires and laboratory assessments. Variables included age, race/ethnicity, and educational attainment. Smoking behavior was categorized as current smoking, exposure to Environmental Tobacco Smoke (ETS), or non-smoking, based on questionnaire responses combined with serum cotinine levels.^17^ Alcohol intake was evaluated by self-reported consumption of at least 12 drinks within the past year. Body Mass Index (BMI) was calculated as weight in kilograms divided by height in meters squared.

Clinical conditions were defined according to standardized criteria. Hypertension was identified by either self-reported antihypertensive treatment or an average blood pressure ≥140/90 mmHg. Diabetes mellitus was defined as fasting glucose ≥126 mg/dL, HbA1c ≥6.5%, or reported use of antidiabetic medications. Hypercholesterolemia was determined by fasting LDL cholesterol ≥130 mg/dL or current lipid-lowering therapy. Kidney function was assessed using the 2021 CKD-EPI creatinine-cystatin C equation,^18^ with Chronic Kidney Disease (CKD) defined as estimated glomerular filtration rate based on both serum creatinine and cystatin C (eGFRcr-cys) < 60 mL/min/1.73 m^2^ or Urine Albumin-to-Creatinine Ratio (UACR) ≥ 30 mg/g.^19^ C-Reactive Protein (CRP) levels were measured by latex-enhanced nephelometry, in which plasma samples react with latex particles coated with anti-CRP antibodies to form antigen–antibody complexes, and the scattered light intensity is quantified against calibration standards.^20^

Comorbidities were identified through the medical history questionnaire. Cardiovascular Disease (CVD) history was defined by self-reported physician diagnoses of heart failure, coronary heart disease, angina pectoris, myocardial infarction, or stroke. Lung disease history included self-reported physician diagnoses of emphysema or chronic bronchitis. Cancer history was determined from self-reported responses collected during household interviews.^21^

### Mortality data linkage

The National Center for Health Statistics (NCHS) has linked NHANES 2001–2002 participants to the National Death Index, enabling long-term follow-up of vital status through 2019. This linkage provides comprehensive information on overall and cause-specific mortality. In the present study, survival status and follow-up time were determined, and deaths were classified into seven categories: all-cause mortality, cardiovascular mortality, diabetes mellitus mortality, chronic lower respiratory disease mortality, Alzheimer’s disease mortality, nephritis/nephrotic syndrome/nephrosis mortality, and cancer mortality. Cardiovascular mortality was defined as deaths attributable to heart disease or cerebrovascular events. For cancer mortality, the publicly available dataset only indicates whether death was cancer-related, without specifying cancer type. Additional details regarding data linkage and analytic procedures are available on the NCHS website.^22^

### Statistics

All analyses incorporated the complex multistage probability sampling design of NHANES, with survey weights, strata, and primary sampling units applied to obtain nationally representative estimates.^23^ Due to skewed distributions, HE4, eGFRcr-cys, UACR, and CRP were natural log-transformed (ln). Survey-weighted geometric means of HE4 and 95% Confidence Intervals (95% CI), back-transformed from ln-HE4 values, were calculated within demographic and clinical subgroups. Differences across subgroups were tested using complex samples general linear models. Baseline age was summarized by vital status. Age at death among deceased participants and age at censoring among survivors were calculated as age at serum collection plus follow-up time in months divided by 12. Survey-weighted means, standard errors, and 95% CI were reported. Associations between ln-HE4 and chronic conditions were examined using survey-weighted logistic regression models. These models were adjusted for age, ethnicity, education, smoking status, alcohol intake, and BMI. Survey-weighted linear regression was used to examine the associations of continuous covariates with ln-HE4.

For mortality outcomes, survey-weighted Cox proportional hazards models were fitted to estimate Hazard Ratios (HR) and 95% CI. Model 1 adjusted for age, ethnicity, education, smoking status, alcohol intake, and BMI. Model 2 further adjusted for hypertension, hypercholesterolemia, diabetes mellitus, and CRP. Model 3 further adjusted for eGFRcr-cys and UACR. The proportional hazards assumption was assessed using the test implemented in the complex samples Cox regression procedure. Nonlinearity was evaluated by adding quadratic and cubic terms for ln-HE4. Effect modification was examined by including cross-product terms between ln-HE4 and prespecified demographic and clinical variables. Interaction analyses were exploratory, and no correction for multiple testing was applied to the interaction p-values. For nominally significant interactions involving continuous UACR or eGFRcr-cys, we additionally estimated stratum-specific HRs using clinically relevant thresholds: UACR < 30 or ≥ 30 mg/g and eGFRcr-cys ≥ 60 or < 60 mL/min/1.73 m^2^. For categorical analyses, HR across HE4 quartiles were estimated using the same design-adjusted Cox framework. Dose-response trends were tested by entering quartile rank as an ordinal continuous variable and using the Wald test. Model-based survival curves by HE4 quartile were also generated from the design-weighted Cox models.

We further performed four sensitivity analyses. First, in exclusion analyses, participants with baseline cardiovascular disease, cancer, or lung disease were removed, and the associations were re-estimated using the Model 3 adjustment set. Second, to reduce potential reverse causation, we performed outcome-specific early-death exclusion analyses. For all-cause mortality, deaths from any cause within the first 24-months were excluded. For cause-specific mortality, only deaths from the corresponding cause within the first 24-months were excluded; early deaths from other causes were retained as non-events. Models were re-estimated using the Model 3 adjustment set. Third, we fitted a history-adjusted model based on Model 3 with additional adjustment for baseline history of cardiovascular disease, lung disease, and cancer (Model 4). Because baseline diseases could lie on the causal pathway between HE4 and mortality, the history-adjusted model was reported in the Supplement to minimize potential overadjustment in the main results. Fourth, to further address potential confounding by age, we repeated Cox proportional hazards models using attained age as the time scale. Age at serum collection was used as the entry time, and age at death or censoring was used as the exit time. Because age was incorporated directly into the time scale, baseline age was not additionally included as a covariate in this sensitivity model; other covariates were kept consistent with the fully adjusted model.

A two-tailed p-value < 0.05 was considered statistically significant. All analyses were performed using SPSS version 30.0 (IBM Corp., Armonk, NY, USA), except for the attained-age time-scale sensitivity analysis, which was performed in R using the survey and survival packages.

## Results

### Baseline characteristics and distribution of HE4

The participants had a mean age of 46.2-years (range 20–85) based on the complex sampling design. Serum HE4 levels were detectable in all individuals, with a mean of 19.50 picomolar (range: 0.38–312.08). Over a median follow-up of-199.3-months, 466 deaths occurred, including 122 attributed to cardiovascular disease, 93 to cancer, 29 to Alzheimer’s disease, 25 to chronic lower respiratory disease, 18 to diabetes mellitus, and 13 to nephritis/nephrotic syndrome/nephrosis.

As shown in Table 1, the overall survey-weighted geometric mean of serum HE4 was 14.79 picomolar (95% CI: 13.98–15.64). HE4 levels increased with age, from 11.13 picomolar (95% CI: 10.48–11.82) in participants aged 20–39 years to 25.20 picomolar (95% CI: 23.80–26.68) in those aged ≥ 60 years (p < 0.001). Higher levels were also found among individuals with lower education, non-Hispanic white, current smokers, and those with hypertension, diabetes, hypercholesterolemia, history CVD, history of cancer, history of lung disease, elevated UACR, reduced eGFRcr-cys, or higher CRP. Because serum HE4 increased substantially with age, we additionally summarized age according to vital status. In the Supplementary Table 1, deceased participants were older at serum collection than non-deceased participants (survey-weighted mean ± SE, 66.69 ± 1.15 vs. 41.86 ± 0.46 years; design-based mean difference, 24.83-years; 95% CI 21.98–27.68; p < 0.001). Age at death among deceased participants, calculated as baseline age plus follow-up months/12, was 76.89 ± 1.09 years. For non-deceased participants, the corresponding age at censoring was 59.86 ± 0.47 years.

**Table 1:** Survey-weighted geometric mean and 95% CI of HE4 in the study population (unweighted n = 2200; weighted population 46,904,119).

		HE4 (picomolar)			
		n	Geometric means	95% CI	p-value
**Total**		2200	14.79	13.98–15.64	
Age (years)	20‒39	902	11.13	10.48–11.82	<0.001
	40‒59	636	14.44	13.35–15.61	
	≥ 60	662	25.20	23.80–26.68	
Ethnicity	Mexican-American	467	11.86	10.38–13.54	0.035
	Other Hispanic	90	12.69	9.28–17.35	
	Non-Hispanic white	1173	15.70	14.74–16.73	
	Non-Hispanic black	390	12.62	11.34–14.05	
	Other ethnicity	80	12.98	9.98–16.87	
Education level	< 9th	268	17.04	15.08–19.25	<0.001
	9–11th	355	18.4	16.28–20.80	
	HS/GED	530	15.89	14.41–17.52	
	SomeCol/AA	629	13.68	12.70–14.75	
	≥ College	418	13.05	12.32–13.82	
BMI (kg/m^2^)	< 25	735	15.14	14.15–16.19	0.449
	25–30	714	14.82	13.38–16.42	
	> 30	751	14.35	13.42–15.34	
Smoking status	Non-smoker	1337	13.76	12.91–14.66	<0.001
	ETS	386	13.47	12.25–14.81	
	Current smoker	477	18.53	16.94–20.28	
Alcohol consumption (drinks/year)	< 12	1061	15.68	14.41–17.06	0.025
	≥ 12	1139	14.2	13.32–15.13	
Hypertension	No	1449	13.14	12.35–13.99	<0.001
	Yes	751	19.22	18.07–20.44	
Diabetes Mellitus	No	1962	14.37	13.55–15.24	<0.001
	Yes	238	20.72	18.65–23.02	
Hypercholesterolemia	No	1654	13.64	12.94–14.38	<0.001
	Yes	546	19.31	17.48–21.33	
History of CVD	No	2032	14.2	13.47–14.97	<0.001
	Yes	168	25.45	19.20–33.75	
History of cancer	No	2005	14.36	13.60–15.16	<0.001
	Yes	195	19.18	16.97–21.68	
History of lung disease	No	2029	14.39	13.58–15.29	<0.001
	Yes	171	19.63	17.65–21.71	
UACR (mg/g)	< 30	1944	14.31	13.50–15.17	<0.001
	≥ 30	256	20.17	16.90–24.06	
eGFRcr-cys (mL/min/1.73 m^2^)	≥ 60	2042	13.83	13.15–14.54	<0.001
	< 60	158	45.71	41.30–50.59	
CRP (mg/dL)	< 0.1	465	13.49	12.46–14.61	0.013
	≥ 0.1	1735	15.28	14.31–16.32	

Group differences were tested using Complex Samples General Linear Models.

BMI, Body Mass Index; CRP, C-Reactive Protein; CVD, Cardiovascular Disease; eGFRcr-cys, Estimated Glomerular Filtration Rate based on both serum creatinine and cystatin-C; ETS, Environmental Tobacco Smoker; HE4, Human Epididymal Secretory protein-E4; UACR, Urine Albumin-to-Creatinine Ratio.

### Associations with chronic diseases

Survey-weighted logistic regression results are presented in Table 2. Higher HE4 levels were strongly associated with CKD (OR = 2.07, 95% CI: 1.38–3.12, p = 0.002) and history of lung disease (OR = 1.34, 95% CI: 1.056–1.709, p = 0.019). A marginal association was observed for hypercholesterolemia (OR = 1.28, 95% CI: 1.00–1.65, p = 0.050), while no significant associations were detected for hypertension, diabetes, CVD, or cancer.

**Table 2:** OR (95% Confidence Interval [95% CI]) of chronic diseases with one unit increase in ln- HE4 in complex samples of logistic regression analysis, with results weighted for sampling strategy (unweighted n = 2200; weighted population 46,904,119).

	Ln-HE4 (picomolar)	
	OR (95% CI)	p-value
**Hypertension**	0.89 (0.68‒1.17)	0.368
**Diabetes Mellitus**	1.22 (0.90‒1.64)	0.191
**Hypercholesterolemia**	1.28 (1.00‒1.65)	0.050
Chronic kidney disease*	2.07 (1.38‒3.12)	0.002
**History of CVD**	1.28 (0.66‒2.47)	0.444
**History of cancer**	1.09 (0.84‒1.44)	0.480
**History of lung disease**	1.34 (1.06‒1.71)	0.019

Adjusted for Model 1: Age, ethnicity, education, smoking, drinking, and BMI.

BMI, Body Mass Index; CI, Confidence Intervals; CVD, Cardiovascular Diseases; HE4, Human Epididymal Secretory Protein E4; OR, Odds Ratios. Chronic kidney disease: eGFRcr-cys < 60 mL/min/1.73 m^2^ or a urine albumin-to- UACR ≥ 30 mg/g.

In survey-weighted linear regression analyses (Table 3), both age (β = 9.98, p < 0.001) and ln-UACR (β = 0.11, p = 0.038) were positively associated with ln-HE4 levels, whereas BMI (β = −0.82, p = 0.012) and ln-eGFRcr-cys (β = −0.08, p < 0.001) showed inverse associations. Ln-CRP was not significantly related to ln-HE4.

**Table 3:** Survey-weighted linear regression coefficients (SE) continuous covariates with one unit increase in ln- HE4 in complex samples of multiple linear regression analysis (unweighted n = 2200; weighted population 46,904,119).

	Ln-HE4 (picomolar)	
	β coeff (S.E.)	p-value
**Age (years)**	9.98 (0.49)	<0.001
**BMI (kg/m^2^)**	−0.82 (0.29)	0.012
**Ln-eGFRcr-cys (mL/min/1.73 m^2^)**	−0.08 (0.01)	<0.001
**Ln-CRP (mg/dL)**	0.03 (0.04)	0.557
**Ln-UACR (mg/g)**	0.11 (0.05)	0.038

Adjusted for Model 1: age, ethnicity, education, smoking, drinking, and BMI.

BMI, Body Mass Index; CRP, C-Reactive Protein; eGFRcr-cys, Estimated Glomerular Filtration Rate based on both serum creatinine and cystatin-C; HE4, Human Epididymal secretory protein E4; UACR, Urine Albumin-to-Creatinine Ratio.

Note: When age or BMI served as the dependent variable, they were not included as covariates in the adjusted model.

### Associations with mortality

As shown in Table 4 and Supplementary Table 2, higher ln-HE4 levels were associated with increased risks of several mortality outcomes, some of which remained statistically significant after full adjustment. In the fully adjusted model (Model 3), each unit increase in ln-HE4 was associated with a significantly higher risk of all-cause mortality (HR = 1.29, 95% CI: 1.03–1.63, p = 0.031). For cancer-related mortality, the association also remained significant (HR = 1.60, 95% CI: 1.02–2.49, p = 0.041). A particularly strong association was observed for chronic lower respiratory disease mortality, with an approximately threefold increase in risk (HR = 3.16, 95% CI: 1.26–7.91, p = 0.017). The association with diabetes-related mortality showed an elevated point estimate (HR = 3.84, 95% CI: 0.92–16.00, p = 0.063), but did not reach statistical significance after full adjustment. In contrast, ln-HE4 was not significantly associated with Alzheimer’s disease mortality, cardiovascular mortality, or nephritis/nephrotic syndrome/nephrosis mortality. The proportional hazards assumption was formally evaluated using the test implemented in the complex samples Cox regression procedure. No significant violation was observed in estimable models, whereas formal testing was not estimable for Alzheimer’s disease mortality and nephritis/nephrotic syndrome/nephrosis mortality because of sparse events. For outcomes with significant associations, we tested nonlinearity by adding quadratic and cubic terms; the higher-order terms were not significant, indicating that a linear specification best described these relationships.

**Table 4:** HR (95% CI) for mortality outcomes associated with a unit increase in ln-HE4. Results are derived from a weighted Cox regression model accounting for complex sampling design (unweighted n = 2200; weighted population 46,904,119).

	HE4 (picomolar)	
	HR (95% CI)	p-value
**All-cause mortality**		
Model 1	1.61 (1.35‒1.92)	<0.001
Model 2	1.57 (1.31‒1.88)	<0.001
Model 3	1.29 (1.03‒1.63)	0.031
**Cancer mortality**		
Model 1	1.44 (1.02‒2.04)	0.039
Model 2	1.43 (1.03‒1.99)	0.037
Model 3	1.60 (1.02‒2.49)	0.041
**Chronic lower respiratory disease mortality**		
Model 1	2.33 (1.30‒4.17)	0.008
Model 2	2.06 (1.08‒3.94)	0.030
Model 3	3.16 (1.26‒7.91)	0.017
**Diabetes mellitus mortality**		
Model 1	5.40 (1.54‒18.87)	0.012
Model 2	2.65 (0.81‒8.66)	0.100
Model 3	3.84 (0.92‒16.00)	0.063

Model 1 adjusted for age, ethnicity, education, smoking, drinking, and BMI. Model 2 adjusted for Model 1 plus hypertension, hypercholesterolemia, diabetes mellitus, and CRP. Model 3 adjusted for Model 2 plus eGFRcr-cys, UACR, HR are shown per one-unit increase in natural log-transformed serum HE4. All models accounted for NHANES survey weights, strata, and primary sampling units.

BMI, Body Mass Index; CRP, C-Reactive Protein; CVD, Cardiovascular Disease; eGFRcr-cys, Estimated Glomerular Filtration Rate based on both serum creatinine and cystatin-C; HE4, Human Epididymal Secretory Protein E4; HR, Hazard Ratios; UACR, Urine Albumin-to-Creatinine Ratio.

Exploratory interaction analyses are presented in Supplementary Table 3. No correction for multiple testing was applied. Nominally significant interactions were observed for ln-HE4 with continuous UACR in relation to cancer mortality and chronic lower respiratory disease mortality, and with continuous eGFRcr-cys in relation to diabetes mortality. To aid interpretation, stratum-specific HRs based on clinically relevant thresholds are provided in Supplementary Table 4. In stratified analyses for nominal interaction findings, ln-HE4 showed a stronger association with cancer mortality among participants with UACR ≥ 30 mg/g (HR = 2.83, 95% CI: 1.34–5.97) than among those with UACR < 30 mg/g (HR = 1.37, 95% CI: 0.93–2.01; continuous UACR interaction p = 0.011). For chronic lower respiratory disease mortality, estimates were positive across UACR strata but imprecise, particularly in the UACR ≥ 30 mg/g group with very few events (n = 3; interaction p = 0.002). For diabetes mortality, the association appeared stronger among participants with eGFRcr-cys ≥ 60 mL/min/1.73 m^2^ (HR = 4.15, 95% CI: 1.13–15.28) than among those with eGFRcr-cys < 60 mL/min/1.73 m^2^ (HR = 1.80, 95% CI: 0.64–5.04; interaction p = 0.021). These exploratory interaction findings should be interpreted cautiously.

**Fig. 2:**
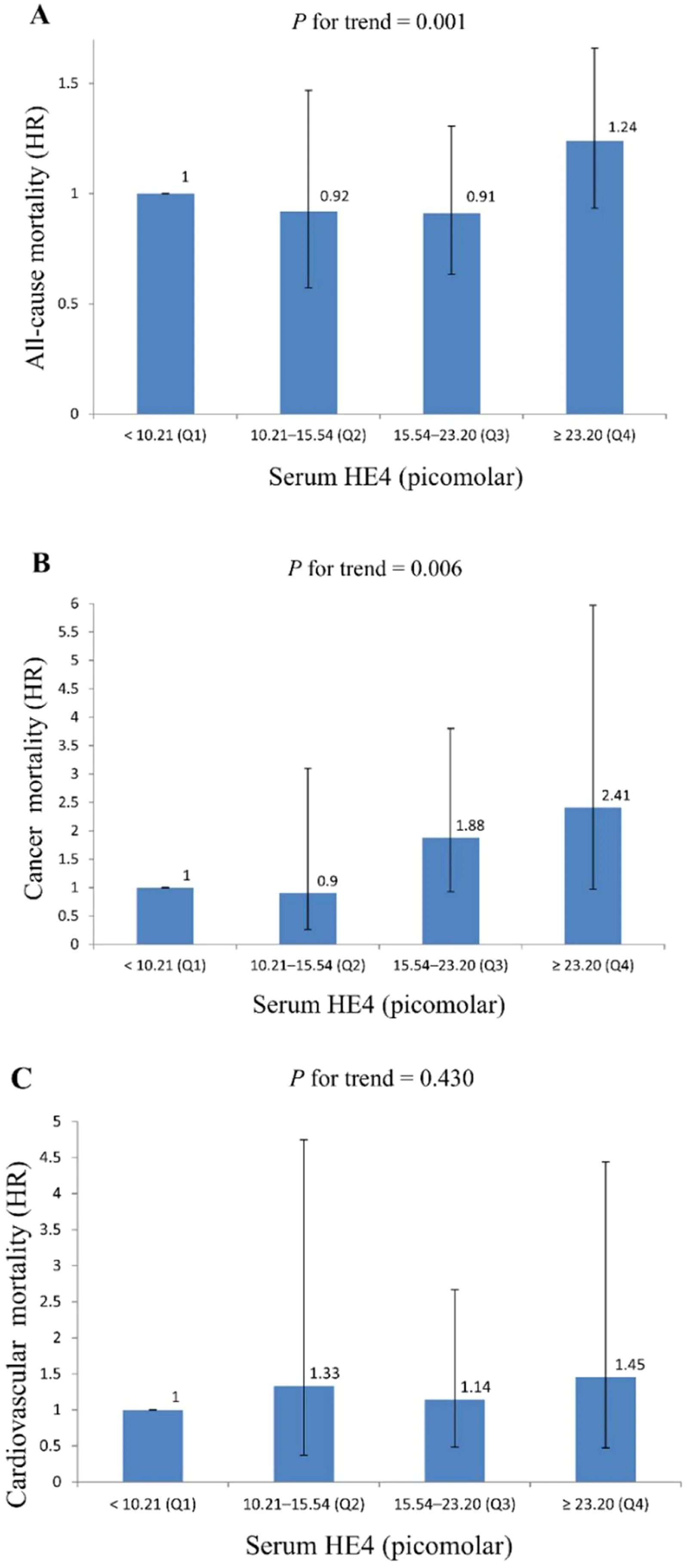
Survey-weighted HR (95% CI) across serum HE4 quartiles from Cox models (adjusted for Model 3, including age, ethnicity, education, smoking status, alcohol intake, BMI, hypertension, hypercholesterolemia, diabetes mellitus, CRP, eGFRcr-cys, and UACR). Panels: (A) All-cause mortality; (B) Cancer mortality; (C) Cardiovascular mortality.

**Fig. 3:**
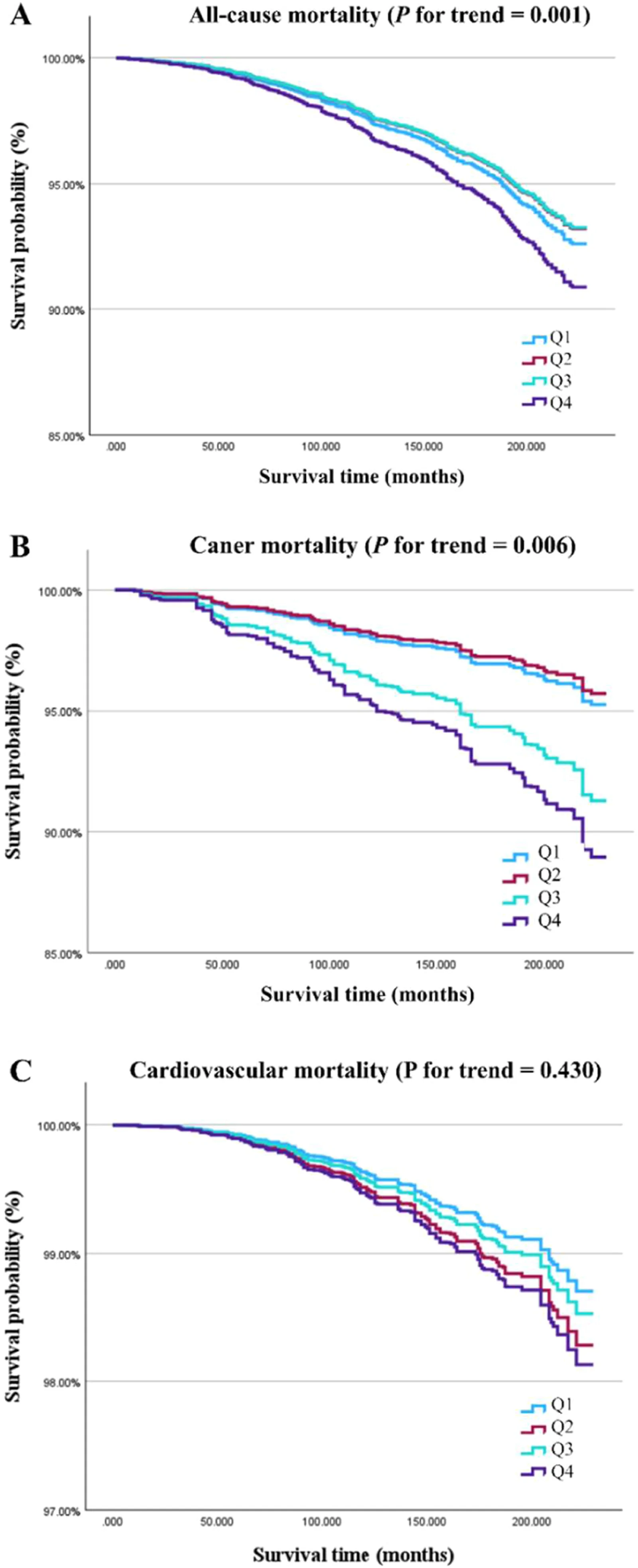
Design-adjusted survival curves by serum HE4 quartile from the fully adjusted Cox model (adjusted for Model 3, including age, ethnicity, education, smoking status, alcohol intake, BMI, hypertension, hypercholesterolemia, diabetes mellitus, CRP, eGFRcr-cys, and UACR). Panels: (A) All-cause mortality; (B) Cancer mortality; (C) Cardiovascular mortality.

Fig. 2 illustrate the dose–response trends across quartiles of serum HE4 levels in relation to cause-specific mortality outcomes. Dose-response trends for Alzheimer’s disease, chronic lower respiratory disease, diabetes, and nephritis/nephrotic syndrome/nephrosis mortality were not evaluated due to limited events and are therefore not shown. Survey-weighted Cox models (Model 3) showed that higher HE4 quartiles were associated with greater risks of all-cause (p for trend = 0.001) and cancer mortality (p for trend = 0.006), with no significant trend for cardiovascular mortality. Fig. 3 presents the corresponding design-adjusted survival curves from the same model, which show progressive separation across HE4 quartiles for all-cause and cancer mortality, but minimal separation for cardiovascular mortality, consistent with the HR plots.

### Sensitivity analysis

In sensitivity analyses (Table 5) using survey-weighted Cox models (Model 3) and excluding participants with baseline CVD, cancer, or lung disease, the positive associations of ln-HE4 with all-cause and cancer mortality were generally robust but attenuated under some exclusions (all-cause mortality after excluding CVD; cancer mortality after excluding cancer; chronic lower respiratory disease mortality after excluding cancer and lung disease). However, estimates for chronic lower respiratory disease mortality were inconsistent and imprecise. In sensitivity analyses, associations with diabetes mortality emerged in the CVD- and lung-disease–exclusion subsets. In the outcome-specific early-death exclusion analyses, the associations of ln-HE4 with all-cause mortality, chronic lower respiratory disease mortality, and cancer mortality remained statistically significant. Specifically, for all-cause mortality, participants who died from any cause within the first 24-months were excluded; for cause-specific mortality outcomes, only participants who died from the corresponding cause within the first 24-months were excluded. After these exclusion, the associations of ln-HE4 with all-cause mortality (HR = 1.56, 95% CI: 1.26–1.93), chronic lower respiratory disease mortality (HR = 3.48, 95% CI: 1.20–10.04), and cancer mortality (HR = 1.55, 95% CI: 1.01–2.39) remained statistically significant, whereas the association with diabetes mortality was attenuated and no longer statistically significant, although the point estimate remained elevated (HR = 5.94, 95% CI: 0.79–44.80). For other outcomes (Supplementary Table 5), ln-HE4 showed no consistent associations with cardiovascular or nephritis/nephrotic syndrome/nephrosis mortality across exclusions. After excluding deaths within the first 2-years of follow-up, the associations with Alzheimer’s disease, cardiovascular, and nephritis/nephrotic syndrome/nephrosis mortality also remained non-significant. An apparent association with Alzheimer’s disease was observed only in the cancer-exclusion analysis.

**Table 5:** HR (95% CI) for all-cause, chronic lower respiratory disease, diabetes mellitus, and cancer mortality associated with a unit increase in ln-HE4. Results are derived from a weighted Cox regression model accounting for complex sampling design.

	HE4 (picomolar)	
	HR (95% CI)	p-value
**Exclude subjects with a history of CVD (n = 2032)**		
All-cause mortality	1.25 (0.97‒1.63)	0.082
Chronic lower respiratory disease mortality	3.24 (1.01‒10.43)	0.049
Diabetes mellitus mortality	45.25 (5.55‒369.01)	0.002
Cancer mortality	1.62 (1.04‒2.55)	0.034
**Exclude subjects with a history of cancer (n = 2005)**		
All-cause mortality	1.29 (1.06‒1.58)	0.015
Chronic lower respiratory disease mortality	2.31 (0.64‒8.26)	0.184
Diabetes mellitus mortality	3.11 (0.79‒12.22)	0.097
Cancer mortality	1.64 (0.88‒3.08)	0.112
**Exclude subjects with a history of lung disease (n = 2029)**		
All-cause mortality	1.32 (1.12‒1.56)	0.003
Chronic lower respiratory disease mortality	1.12 (0.35‒3.57)	0.840
Diabetes mellitus mortality	10.48 (1.60‒68.75)	0.018
Cancer mortality	1.59 (1.01‒2.50)	0.045
**Excluding deaths within the first 2-years of follow-up**		
All-cause mortality (n = 2178)	1.56 (1.26‒1.93)	<0.001
Chronic lower respiratory disease mortality (n = 2198)	3.48 (1.20‒10.04)	0.024
Diabetes mellitus mortality (n = 2199)	5.94 (0.79‒44.80)	0.080
Cancer mortality (n = 2194)	1.55 (1.01‒2.39)	0.047

Adjusted for Model 3, including age, ethnicity, education, smoking status, alcohol intake, BMI, hypertension, hypercholesterolemia, diabetes mellitus, CRP, eGFRcr-cys, and UACR. HR are shown per one-unit increase in ln-HE4. All models accounted for NHANES survey weights, strata, and primary sampling units. Early-death exclusion was outcome-specific.

CVD, Cardiovascular Disease; HE4, Human Epididymal secretory protein-E4; HR, Hazard Ratios.

*Cardiovascular mortality: Death from heart or cerebrovascular disease.

In the history-adjusted model, Supplementary Table 6 shows that a one-unit increase in ln-HE4 was associated with higher risks of all-cause, cancer, and diabetes mortality in Model 4 (survey-weighted Cox regression). The association with chronic lower respiratory disease mortality was weaker and did not reach statistical significance, while no significant associations were observed for cardiovascular, Alzheimer’s disease, or nephritis/nephrotic syndrome/nephrosis mortality.

To further address potential confounding by age, we repeated the Cox models using attained age as the time scale, with age at serum collection as the entry time and age at death or censoring as the exit time. In the Supplementary Table 7, the associations of ln-HE4 with all-cause mortality, cancer mortality, and chronic lower respiratory disease mortality remained materially unchanged. The corresponding HRs were 1.26 (95% CI 1.01–1.58; p = 0.044), 1.58 (95% CI 1.02–2.43; p = 0.040), and 3.35 (95% CI 1.39–8.10; p = 0.010), respectively.

## Discussion

In this nationally representative cohort of U.S. women, higher serum HE4 was associated with greater risks of all-cause and cancer mortality after multivariable adjustment. Chronic lower respiratory disease mortality also showed a positive association, but the estimate was imprecise because of limited events. The estimate for diabetes mortality was imprecise and not statistically significant; therefore, it should be interpreted as inconclusive rather than supportive evidence of an association. Cardiovascular, Alzheimer’s disease, and nephritis-related mortality showed no statistically significant associations. This pattern suggests that HE4 may reflect systemic processes such as fibrotic remodeling or inflammatory burden that relate more closely to oncologic and respiratory pathways than to atherosclerotic events. The presence of dose-response across quartiles, the adequacy of a linear specification, and robustness in sensitivity analyses together argue against baseline comorbidity as the sole explanation. However, caution is needed in interpreting findings with limited event numbers. These findings extend patient-focused evidence to a population setting and support a broader prognostic role for HE4 beyond gynecologic oncology in U.S. women.

In design-weighted logistic models of our study, HE4 was positively associated with chronic kidney disease and with a history of lung disease. These findings align with prior work showing that HE4 is upregulated in fibrotic kidneys and rises as kidney function declines, and that experimental inhibition of HE4 attenuates renal fibrosis.^8^^,^^24^ In the respiratory system, HE4 has been implicated in airway inflammation and remodeling in COPD, providing a mechanistic basis for the observed association with lung disease history.^10^ The associations between HE4 and hypertension, diabetes, clinical CVD, and cancer were null and the association with hypercholesterolemia was borderline. Prior reports demonstrating HE4 as a predictor were conducted in patients with established or advanced disease, where fibrosis and extracellular matrix remodeling are common and drive higher HE4.^12^^,^^13^ Unlike prior patient-based studies, our cross-sectional NHANES analysis focused on the prevalence of upstream CVD risk factors and self-reported disease history at baseline, which HE4 may not capture as well as downstream tissue injury. The null association with cancer history is also plausible because NHANES records “ever cancer” without tumor type, activity, treatment, or timing, so remission and heterogeneity dilute signals. By contrast, our prospective results linking higher HE4 to mortality are consistent with HE4 functioning primarily as a prognostic marker of ongoing fibrotic and remodeling biology.

The positive associations of ln-HE4 with cancer and chronic lower respiratory disease mortality in fully adjusted models are biologically and clinically plausible. For cancer, our findings are consistent with the extensive oncology literature in which HE4 is a prognostic marker in gynecologic malignancies and related settings.^3^^,^^5^ This current study extends this evidence to a population-based cohort. A key limitation is that the NHANES public-use mortality files classify cancer deaths only at the broad level of malignant neoplasms, so tumor site and phenotype could not be examined. For chronic lower respiratory disease mortality, the association aligns with mechanistic data showing that HE4 promotes airway inflammation and remodeling, processes central to chronic obstructive pulmonary disease and related diseases encompassed by the chronic lower respiratory disease category.^10^ Together, these results suggest that HE4 may reflect fibrotic and tissue-remodeling biology relevant to oncologic and respiratory mortality at the population level.

Several disease-cohort studies have linked higher HE4 to adverse cardiovascular outcomes, including cardiovascular death and heart-failure readmission in acute or chronic heart failure and ischemic cardiomyopathy.^11^^,^^12^ In contrast, we observed no significant association with cardiovascular mortality in a community sample of women. This difference likely reflects population and phenotype: prior studies enrolled patients with established, often advanced, cardiac disease in whom myocardial fibrosis, congestion, and renal impairment are common and strongly elevate HE4, whereas NHANES captures a broad population with heterogeneous causes of cardiovascular death where atherosclerotic mechanisms may dominate and be less tightly coupled to HE4 biology. Notably, in a prospective abdominal aortic aneurysm cohort, the association between higher HE4 and cardiovascular death was significant in unadjusted analyses but lost significance after multivariable adjustment, highlighting context-dependent effects.^25^ Limited statistical power, even with 122 cardiovascular deaths, together with reliance on a single baseline HE4 measurement, may also have attenuated the association in our study.

In sensitivity analyses, HE4 was associated with Alzheimer’s disease mortality only when participants with a baseline history of cancer were excluded. This pattern may reflect competing risk and selective survival, whereby earlier cancer deaths preclude Alzheimer’s deaths. Given limited power and possible misclassification, the finding should be interpreted cautiously and requires replication in larger cohorts.

We observed effect modification by albuminuria for cancer and chronic lower respiratory disease mortality. Albuminuria reflects glomerular injury and systemic endothelial dysfunction with microvascular inflammation,^26^ whereas HE4 reflects protease inhibition and fibrotic extracellular matrix remodeling.^7^ These pathways converge in tumor progression through angiogenesis, matrix remodeling, and immune evasion, and in chronic lung disease where airway remodeling and parenchymal fibrosis on a background of chronic low-grade inflammation drive functional decline.^2^ Co-elevation of UACR and HE4 may therefore mark a state in which ongoing injury and maladaptive repair coexist, yielding stronger associations with cancer and respiratory mortality than with atherosclerotic cardiovascular outcomes. We also observed a significant ln-HE4 × eGFRcr-cys interaction for diabetes mortality. HE4 is upregulated in renal fibrosis and increases as kidney function declines, implying both heightened production and reduced clearance.^8^ The lower eGFR reflects a pro-fibrotic, inflammatory milieu that amplifies HE4-related risk. The interaction was most evident for diabetes mortality, possibly because diabetic kidney disease combines hyperglycemia-driven injury with tubulointerstitial fibrosis and systemic inflammation, aligning with HE4 biology.^27^ However, diabetes deaths were few, and further studies are needed to confirm this hypothesis. These interaction findings should be interpreted cautiously because the analyses were exploratory, multiple interaction tests were performed without multiplicity correction, and several cause-specific outcomes had limited event counts. Independent replication is needed before drawing firm conclusions regarding effect modification by albuminuria or kidney function.

Strengths of this study include the use of a nationally representative cohort of U.S. women with appropriate incorporation of NHANES weights, strata, and primary sampling units; long follow up through 2019 with National Death Index linkage; high quality HE4 assays with documented quality control; and a rigorous, prespecified analytic strategy with progressive adjustment, dose response testing, nonlinearity checks, model-based survival curves, and multiple sensitivity analyses. We also examined multiple causes of death, permitting assessment of outcome specificity. Limitations include the observational design and potential residual confounding; reliance on a single baseline HE4 measurement with no repeated measures or time-updated covariates; restriction to women, limiting generalizability; small numbers of events for several outcomes, reducing precision; coarse cause-of-death coding (e.g., no cancer subtypes); possible overadjustment when adjusting for baseline diseases; exclusions due to missing covariates that may introduce selection bias; and the lack of an external validation cohort. In addition, because incremental predictive performance beyond established risk factors was not evaluated, the clinical utility of HE4 for risk prediction should be interpreted cautiously.

## Conclusions

In a nationally representative cohort of U.S. women, higher serum HE4 was independently associated with increased risks of all-cause and cancer mortality after comprehensive adjustment. A positive association was also observed for chronic lower respiratory disease mortality, but this finding was based on few events and should be interpreted cautiously. The diabetes mortality estimate was imprecise and not statistically significant. These findings suggest that serum HE4 may have potential as a population-level prognostic marker beyond oncology. However, its incremental predictive value and clinical utility beyond established risk factors remain to be determined. In addition, the observational design, reliance on a single baseline HE4 measurement from 2001 to 2002, limited events for some causes of death, and restriction to women warrant cautious interpretation. Future work should validate these associations in contemporary cohorts that include men, assess serial HE4 measurements, quantify incremental predictive value beyond established risk factors, and clarify mechanisms, particularly in relation to kidney function and albuminuria.

## Authors’ contributions

Y.-W.F. conducted data analysis and manuscript drafting. H.-C.L. and C.W. contributed to statistical consultation and interpretation. C.-Y.L. conceptualized the study, supervised the analysis, and critically revised the manuscript. All authors read and approved the final version.

## Consent for publication

Not applicable.

## Ethics approval and consent to participate

The NHANES protocols for 2001‒2002 were approved by the NCHS Research Ethics Review Board (Protocol #98–12), and all participants provided written informed consent. This secondary analysis used publicly available, de-identified data and was granted exemption from further institutional review by the NCHS Research Ethics Review Board.

## Declaration of generative AI and AI-assisted technologies in the manuscript preparation process

During the preparation of this work, the authors used ChatGPT to assist with language editing and manuscript formatting. After using this tool, the authors reviewed and edited the content as needed and take full responsibility for the content of the publication.

## Funding

This research did not receive any specific grant from funding agencies in the public, commercial, or not-for-profit sectors.

## Conflicts of interest

The authors declare no conflicts of interest.

## Data Availability

All NHANES data are publicly available at https://wwwn.cdc.gov/nchs/nhanes/default.aspx.
