# Supplementary material for: Serum human epididymis protein 4 and long-term mortality in U.S. women: a nationally representative cohort study
Source: Clinics (Sao Paulo). 2026 Sep 11;81:101133. doi: 10.1016/j.clinsp.2026.101133 (PMC13586584; doi:10.1016/j.clinsp.2026.101133)
Supplement: Supplementary file 1 [file mmc1.docx]

**CLINICS-D-25-02176**

**Supplementary material**

**Supplementary Table 1. Age at serum collection and age at death or censoring according to vital status.**

| Vital status at end of follow-up | Unweighted N | Weighted population, % | Age at serum collection, years | Age at death or censoring, years |
| --- | --- | --- | --- | --- |
| Non-deceased | 1,734 | 82.2 | 41.86 ± 0.46 (95% CI, 40.87–42.85) | 59.86 ± 0.47 (95% CI, 58.85–60.87) |
| Deceased | 466 | 17.8 | 66.69 ± 1.15 (95% CI, 64.24–69.15) | 76.89 ± 1.09 (95% CI, 74.56–79.21) |
| Design-based difference (deceased – non-deceased) | — | — | 24.83 (95% CI, 21.98–27.68); P<0.001 | 17.03 (95% CI, 14.48–19.58); P<0.001 |

Notes: Values are survey-weighted means ± SE unless otherwise indicated.

Abbreviations: HE4, human epididymis protein 4.

**Supplementary Table 2.** HR (95% CI) for mortality outcomes associated with a unit increase in ln-HE4. Results are derived from a weighted Cox regression model accounting for complex sampling design. (Unweighted no./population size: 2200/ 46,904,119)

|  | HE4 (picomolar) | |
| --- | --- | --- |
|  | HR (95% CI) | *P* value |
| Alzheimer’s disease mortality |  |  |
| Model 1 | 1.09 (0.41—2.88) | 0.852 |
| Model 2 | 1.22 (0.46—3.23) | 0.675 |
| Model 3 | 1.92 (0.49—7.51) | 0.325 |
| Cardiovascular mortality* |  |  |
| Model 1 | 1.74 (1.17—2.59) | 0.010 |
| Model 2 | 1.71 (1.16—2.53) | 0.010 |
| Model 3 | 1.37 (0.89—2.11) | 0.146 |
| Nephritis/nephrotic syndrome/nephrosis mortality |  |  |
| Model 1 | 8.34 (1.08—64.36) | 0.043 |
| Model 2 | 6.36 (1.08—12.62) | 0.071 |
| Model 3 | 4.49 (0.37—54.96) | 0.221 |

Model 1 adjusted for age, ethnicity, education, smoking, drinking, and BMI.

Model 2 adjusted for model 1 plus hypertension, hypercholesterolemia, diabetes mellitus, and CRP.

Model 3 adjusted for model 2 plus eGFRcr-cys, UACR,

Abbreviations: BMI: body mass index; CRP: C-reactive protein; CVD: cardiovascular disease; eGFRcr-cys: estimated glomerular filtration rate based on both serum creatinine and cystatin C; HE4: human epididymal secretory protein E4; HR: hazard ratios; UACR: urine albumin-to-creatinine ratio.

**Supplementary Table 3.** P values for interaction in the associations of a one-unit increase in ln-HE4 with cause-specific mortality outcomes were derived from survey-weighted Cox proportional hazards models accounting for the complex sampling design and including cross-product terms between ln-HE4 and each covariate.

|  | All-cause mortality | Cancer mortality | Chronic lower respiratory disease mortality | Diabetes mellitus mortality |
| --- | --- | --- | --- | --- |
|  | P for interaction | | | |
| Age (years) | 0.481 | 0.283 | 0.705 | 0.447 |
| Ethnicity | 0.800 | 0.778 | 0.270 | 0.724 |
| Education | 0.076 | 0.152 | 0.968 | 0.944 |
| BMI (kg/m^2^) | 0.676 | 0.913 | 0.565 | 0.563 |
| Smoking status | 0.727 | 0.940 | 0.077 | 0.110 |
| Alcohol consumption (drinks/year) | 0.839 | 0.528 | 0.730 | 0.053 |
| Hypertension | 0.212 | 0.799 | 0.743 | 0.150 |
| Hypercholesterolemia | 0.531 | 0.741 | 0.418 | 0.195 |
| Diabetes mellitus | 0.284 | 0.286 | 0.943 | 0.131 |
| UACR (mg/g) | 0.716 | 0.011 | 0.002 | 0.988 |
| eGFRcr-cys (mL/min/1.73 m²) | 0.960 | 0.914 | 0.396 | 0.021 |
| CRP (mg/dL) | 0.883 | 0.432 | 0.252 | 0.387 |

Adjusted for model 3, including age, ethnicity, education, smoking status, alcohol intake, BMI, hypertension, hypercholesterolemia, diabetes mellitus, CRP, eGFRcr-cys, and UACR.

Abbreviations: BMI: body mass index; CRP: C-reactive protein; eGFRcr-cys: estimated glomerular filtration rate based on both serum creatinine and cystatin C; UACR: urine albumin-to-creatinine ratio.

**Supplementary Table 4.** Stratum-specific associations of ln-HE4 with mortality for nominally significant exploratory interactions

| **Outcome** | **Effect modifier** | **Stratum** | **N** | **Events** | **HR (95% CI)** | **P value** | **P for interaction** |
| --- | --- | --- | --- | --- | --- | --- | --- |
| Cancer mortality | UACR | <30 mg/g | 1944 | 74 | 1.37 (0.93–2.01) | 0.1 | 0.011 |
| Cancer mortality | UACR | ≥30 mg/g | 256 | 19 | 2.83 (1.34–5.97) | 0.009 |  |
| Chronic lower respiratory disease mortality | UACR | <30 mg/g | 1944 | 22 | 3.14 (1.11–8.90) | 0.034 | 0.002 |
| Chronic lower respiratory disease mortality | UACR | ≥30 mg/g | 256 | 3 | 5.52 (1.13–27.12) | 0.037 |  |
| Diabetes mellitus mortality | eGFRcr-cys | ≥60 mL/min/1.73 m² | 2042 | 10 | 4.15 (1.13–15.28) | 0.034 | 0.021 |
| Diabetes mellitus mortality | eGFRcr-cys | <60 mL/min/1.73 m² | 158 | 8 | 1.80 (0.64–5.04) | 0.243 |  |

For UACR-stratified analyses, models were adjusted for age, ethnicity, education, smoking status, alcohol intake, BMI, hypertension, hypercholesterolemia, diabetes mellitus, CRP, and eGFRcr-cys. For eGFRcr-cys-stratified analyses, models were adjusted for age, ethnicity, education, smoking status, alcohol intake, BMI, hypertension, hypercholesterolemia, diabetes mellitus, CRP, and UACR.Abbreviations: CI, confidence interval; eGFRcr-cys, estimated glomerular filtration rate based on both serum creatinine and cystatin C; HE4, human epididymis protein 4; HR, hazard ratio; UACR, urine albumin-to-creatinine ratio.

**Supplementary Table 5.** HR (95% CI) for Alzheimer’s disease, cardiovascular, and nephritis/nephrotic syndrome/nephrosis mortality associated with a unit increase in ln-HE4. Results are derived from a weighted Cox regression model accounting for complex sampling design.

|  | HE4 (picomolar) | |
| --- | --- | --- |
|  | HR (95% CI) | *P* value |
| Exclude subjects with a history of CVD (N=2032) |  |  |
| Alzheimer’s disease mortality | 2.32 (0.47—11.50) | 0.281 |
| Cardiovascular mortality^*^ | 1.26 (0.76—2.08) | 0.343 |
| Nephritis/nephrotic syndrome/nephrosis mortality | 5.85 (0.28—123.40) | 0.236 |
| Exclude subjects with a history of cancer (N=2005) |  |  |
| Alzheimer’s disease mortality | 4.15 (1.28—13.45) | 0.021 |
| Cardiovascular mortality^*^ | 1.18 (0.769—1.82) | 0.420 |
| Nephritis/nephrotic syndrome/nephrosis mortality | 4.03 (0.35—46.97) | 0.245 |
| Exclude subjects with a history of lung disease (N=2029) |  |  |
| Alzheimer’s disease mortality | 2.13 (0.42—10.95) | 0.339 |
| Cardiovascular mortality^*^ | 1.33 (0.83—2.11) | 0.214 |
| Nephritis/nephrotic syndrome/nephrosis mortality | 4.31 (0.40—46.21) | 0.210 |
| Excluding deaths within the first 2 years of follow-up |  |  |
| Alzheimer’s disease mortality (N=2200) | 1.92 (0.49—7.51) | 0.325 |
| Cardiovascular mortality^*^ (N=2196) | 1.38 (0.87—2.19) | 0.153 |
| Nephritis/nephrotic syndrome/nephrosis mortality(N=2200) | 4.49 (0.37—54.96) | 0.221 |

Adjusted for model 3, including age, ethnicity, education, smoking status, alcohol intake, BMI, hypertension, hypercholesterolemia, diabetes mellitus, CRP, eGFRcr-cys, and UACR.

Abbreviations: CVD: cardiovascular disease; HE4: human epididymal secretory protein E4; HR: hazard ratios.

^*^Cardiovascular mortality: Death from heart or cerebrovascular disease.

**Supplementary Table 6.** HR (95% CI) for mortality outcomes associated with a unit increase in ln-HE4. Results are derived from a weighted Cox regression model (Model 4) accounting for complex sampling design. (Unweighted no./population size: 2200/ 46,904,119)

|  | HE4 (picomolar) | |
| --- | --- | --- |
|  | HR (95% CI) | *P* value |
| All-cause mortality | 1.33 (1.06—1.66) | 0.018 |
| Chronic lower respiratory diseases mortality | 2.27 (0.98—5.24) | 0.055 |
| Diabetes mellitus mortality | 5.26 (1.95—14.22) | 0.003 |
| Cancer-related mortality | 1.70 (1.07—2.69) | 0.028 |
| Alzheimer’s disease mortality | 1.73 (0.55—5.49) | 0.327 |
| Cardiovascular mortality^*^ | 1.41 (0.87—2.30) | 0.152 |
| Nephritis/nephrotic syndrome/nephrosis mortality | 4.91 (0.40—59.94) | 0.196 |

Model 4 adjusted for Model 3 plus a history of CVD, a history of cancer, and a history of lung disease.

Abbreviations: CVD: cardiovascular disease; HE4: human epididymal secretory protein E4; HR: hazard ratios.

^*^Cardiovascular mortality: Death from heart or cerebrovascular disease.

**Supplementary Table 7.** Sensitivity analysis using attained age as the Cox time scale

| Outcome | Events | Primary model HR (95% CI) | P value | Attained-age model HR (95% CI) | P value |
| --- | --- | --- | --- | --- | --- |
| All-cause mortality | 466 | 1.28 (1.02–1.60) | 0.033 | 1.26 (1.01–1.58) | 0.044 |
| Cancer mortality | 93 | 1.59 (1.03–2.47) | 0.038 | 1.58 (1.02–2.43) | 0.040 |
| Chronic lower respiratory disease mortality | 25 | 2.97 (1.12–7.83) | 0.030 | 3.35 (1.39–8.10) | 0.010 |
| Cardiovascular mortality | 122 | 1.28 (0.85–1.94) | 0.218 | 1.25 (0.81–1.91) | 0.291 |
| Diabetes mellitus mortality | 18 | 2.35 (0.78–7.05) | 0.119 | 1.86 (0.71–4.86) | 0.187 |
| Alzheimer's disease mortality | 29 | 1.46 (0.32–6.71) | 0.603 | 1.31 (0.26–6.67) | 0.727 |
| Nephritis/nephrotic syndrome/nephrosis mortality | 13 | 2.84 (0.64–12.58) | 0.156 | 2.34 (0.65–8.39) | 0.176 |

**Note.** Values are HR (95% CI) per 1-unit increase in natural log-transformed serum HE4. The primary model used follow-up time as the time scale and adjusted for baseline age. The attained-age model used age as the time scale, with age at serum collection as the entry time and age at death or censoring as the exit time; Because age was incorporated directly into the time scale, baseline age was not additionally included as a covariate in this sensitivity model; other covariates were kept consistent with the Model 3. Abbreviations: CI, confidence interval; HE4, human epididymal secretory protein 4; HR, hazard ratio.
